# Unraveling Histiocytic Disorders: Two Cases From Resource-Limited Settings

**DOI:** 10.7759/cureus.76622

**Published:** 2024-12-30

**Authors:** Mohamed Elbahoty, Nooran Soror, Mona Elnaggar, Samar M Gad, Yasser Eldowik, Dalia I Halwag, Ashraf Elghandour, Ayman Youssef

**Affiliations:** 1 Hematology Unit, Department of Internal Medicine, Alexandria University, Alexandria, EGY; 2 Department of Pathology, University of Alabama at Birmingham, Birmingham, USA; 3 Department of Hematology, Faculty of Medicine, Alexandria University, Alexandria, EGY; 4 Department of Hematology, Alexandria School of Medicine, Alexandria, EGY; 5 Department of Endocrinology, Alexandria School of Medicine, Alexandria, EGY; 6 Department of Pathology, Al-Azhar School of Medicine, Cairo, EGY; 7 Department of Dermatology, Venereology and Andrology, Faculty of Medicine, Alexandria University, Alexandria, EGY; 8 Department of Anesthesiology and Critical Care, Duke University Medical Center, Durham, USA

**Keywords:** immunohistochemistry (ihc), langerhans cell histiocytosis (lch), multisystem disease, relapse refractory disease, rosai-dorfman disease

## Abstract

Histiocytic disorders include a range of uncommon illnesses marked by the buildup of cells that have developed into macrophages, dendritic cells, or monocytes in diverse tissues and organs. Over 100 distinct subtypes have been documented, exhibiting a diverse array of clinical symptoms, presentations, and histologic features that can be confused with other clinical conditions leading to delayed diagnosis. They affect both children and adults, generating a variety of clinical symptoms that can be limited to one position, numerous areas within one system, or affect many systems in the body. We provide our experiences handling two cases of histiocytic disease in underdeveloped countries where targeted treatment choices are unavailable. The first patient was a 32-year-old female with polyuria, skin lesions, and macroadenoma on MRI. Histopathological and immune-histochemical assessment of skin lesions confirmed the diagnosis of Langerhans cell histiocytosis. The patient responded well to steroids and oral and intrathecal methotrexate. The second patient was a 38-year-old male with recurrent skin lesions followed by retro-orbital discomfort and proptosis. The retro-orbital mass was surgically excised followed by a recurrence a few months later. Histopathological and immune-histochemical assessment confirmed the diagnosis of extranodal Rosai-Dorfman disease. He showed a partial response to methotrexate followed by a remarkable response to rituximab. Because of the wide variety of clinical symptoms, the diversity of experts engaged in assessing and treating these patients, and the scarcity of cases accessible, there is still a need to develop an evidence-based technique for evaluating and treating this complicated disease, especially in resource-limited settings.

## Introduction

Histiocytic disorders (HDs) encompass a range of uncommon diseases that are distinguished by the deposition of cells differentiated into macrophages, dendritic cells (DCs), or monocytes in diverse organs and tissues, which were reported in disease registries in an inconsistent approach [1]. Although some information might be missing, the Surveillance, Epidemiology, and End Results (SEER) database did maintain track of high-risk Langerhans cell histiocytosis (LCH). Additional resources, including the UK Histiocytosis Registry, the Belgian Langerhans Cell Histiocytosis Registry, and the 2014-established International Rare Histiocytic Disorders Registry (IRHDR), are useful. Despite the usefulness of these registries for recording sickness experiences, participation is sporadic, and no data are presently available to the public [2].

Mononuclear phagocytes constitute a family of cells that include DCs, monocytes, and macrophages [3]. A histiocyte is a morphological concept that relates particularly to macrophages found in tissues. Macrophages are massive oval-shaped cells that are largely responsible for eliminating apoptotic cells, debris, and infections. DCs are specialized cells that show antigens on major histocompatibility complex molecules and activate dormant T lymphocytes [4].

Over 100 different subtypes of HDs have been identified, with a wide range of clinical symptoms, presentations, and histology. Since its first classification in 1987, much progress has been made in understanding the cellular origins, molecular pathogenesis, and clinical features of HDs [5]. HDs have been conventionally divided into four distinct categories: juvenile xanthogranuloma (JXG), Erdheim-Chester disease (ECD), LCH, and Rosai-Dorfman disease (RDD), also known as Rosai-Dorfman-Destombes disease [2,5]. This classification was updated in 2022 under histiocyte/macrophage neoplasms to add anaplastic lymphoma kinase (ALK)-related histiocytosis and histiocytic sarcoma, besides moving LCH to be included in the distinct category "Langerhans cell neoplasms" that also includes Langerhans cell sarcoma [6].

LCH is characterized by an aberrant increase in the number of myeloid precursors that differentiate into CD1a+/CD207+ cells in the afflicted regions. The illness develops at any stage of life, with various degrees of systemic involvement. While the odds of recovery are excellent, there is a risk of major long-term neurological or endocrine sequelae that may impair one's quality of life [7]. Based on where it manifests, LCH may be classified as either a multisystem (MS) illness, unifocal disease, or single-system multifocal (SS-M) [8,9]. The most often afflicted organs are the bone, pituitary gland, skin, thyroid, lymph nodes, liver, spleen, and bone marrow. The most frequently affected organs are the liver, spleen, and bone marrow [10].

From the molecular aspect, the discovery of the BRAF-V600E mutation in around 50% of patients confirmed LCH malignant genotype. LCH, along with ECD, is classed as part of the "L-group" in the updated classification of histiocytic diseases. The present corpus of knowledge on LCH is mostly comprised of results from pediatric research, with little information accessible on its adult equivalents. The exact incidence of adult LCH is unknown; estimates range from one to one and a half cases per million persons per year [9].

Since then, other gene alterations have been found, including MAP2K1 and MAP3K1. In prior studies, MAP2K1 or BRAF mutations were found in around 90% of adult LCH patients, BRAFindel in 28.8%, and BRAFV600E in only 31.5% of adults, in contrast to pediatric patients. These data suggest a variation in pathophysiology between LCH in children and adults [11].

There is a confirmed link between LCH and other malignancies, with incidence rates ranging from 2.6% in children to 32% in adults. Thirteen different solid malignancies have been related to LCH; in both adults and children, thyroid carcinoma has been found in association with LCH-induced thyroid infiltration, and lung carcinoma has been recorded frequently in adult series. Both Hodgkin and non-Hodgkin lymphomas have been linked to LCH, and they commonly appear in the same lymph nodes. The most often documented hematologic malignancy is acute myeloid leukemia, which usually occurs years after LCH [12].

RDD is a rare kind of histiocytosis that does not include Langerhans cells, and the etiology is unknown. According to the histiocytosis classification of 2016, RDD includes both familial and sporadic variations. These variants may be further divided as classical nodal, extranodal, unclassified, or RDD associated with neoplasia or immunological disease, all of which come within the R category [13].

Every year, around 100 new cases of RDD are reported in the United States, with a frequency of one in every 200,000. Young people (mean age = 20.6 years) are more likely to be affected [14]. Goyal and colleagues studied 64 RDD patients [9]. Only 8% had classic (nodal only) RDD, whereas 92% had extranodal RDD. While 17 patients (27%) had the disease in one place, 47 patients (73%) had the disease in several locations. With a range of 2-79 years, the median diagnostic age was 50 years. Skin and subcutaneous tissue were checked mostly on physical examination and imaging (52%), followed by lymph nodes (33%). Most patients reported subcutaneous nodules on the chest, arm, back, and thighs, which might be solitary or many. Six of the 33 cases (18%) in this group had skin lesions such as rashes or plaques. One of the five pediatric patients had subcutaneous nodules [15].

Recent studies have shown kinase mutations in both nodal and extranodal RDD, apart from cutaneous RDD. These alterations include ARAF, MAP2K1, NRAS 5, and KRAS. A percentage of RDD patients may be clonal since one study detected mutations in KRAS or MAP2K1 in up to 33% of cases [16]. A study of 23 RDD samples indicated that they were BRAF-V600E wild type, which is consistent with the identification of recurrent BRAF-V600E mutations in ECD, a non-LCH condition [9]. In a similar vein, Chakraborty et al. performed whole-exome sequencing on four RDD patients and found no somatic alterations [17].

## Case presentation

Case 1

A 32-year-old female presented with polyuria, nocturia, and polydipsia that were persistent during the last six months. The patient sought medical advice at an endocrinology clinic. During the examination, there was no lymphadenopathy, hepatomegaly, or splenomegaly.

**Figure 1:**
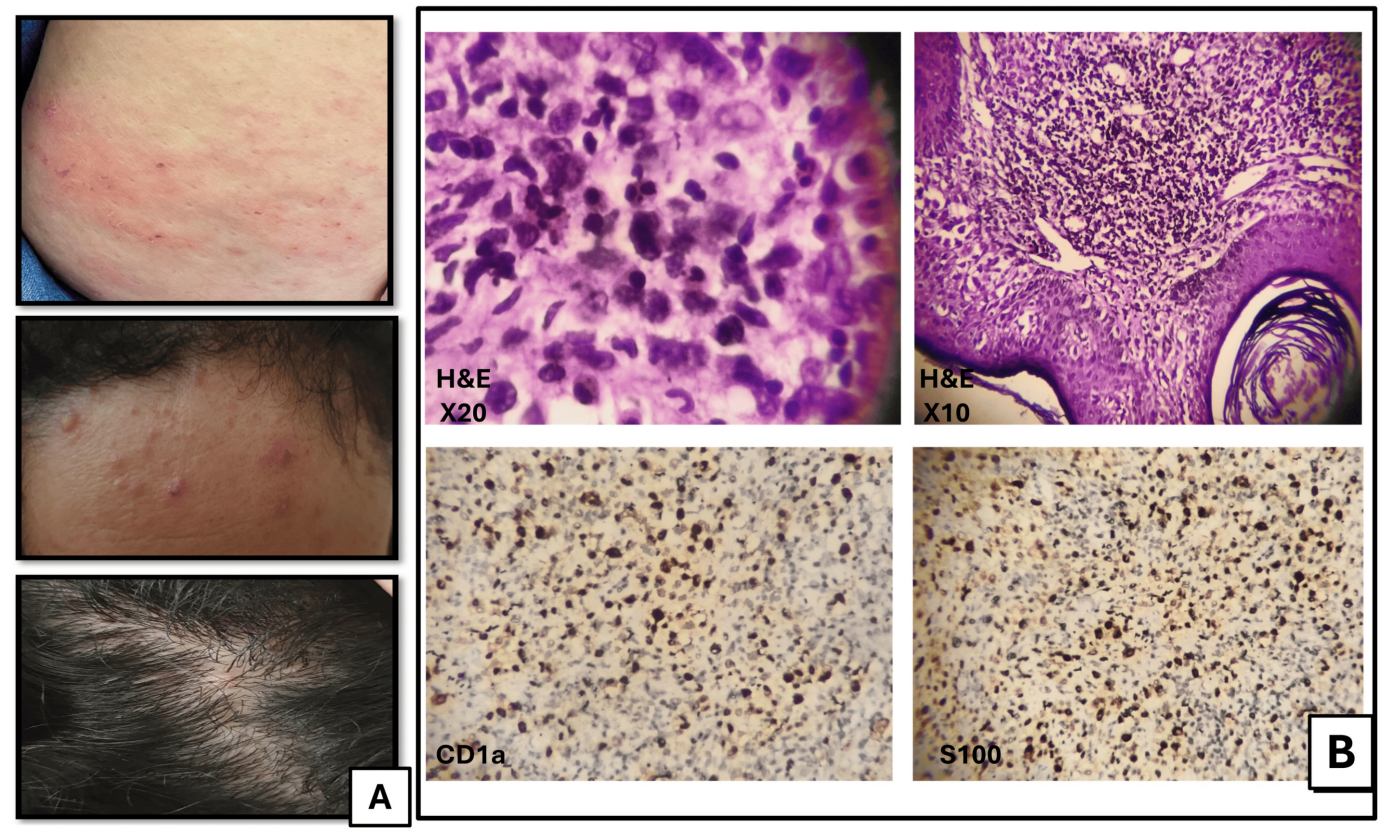
Gross and microscopic pathology of case 1. (A) Multiple, discrete, non-tender, non-scaly, erythematous, non-blanching papules over the scalp, forehead, and breast. (B) The hematoxylin & eosin (H&E) sections (upper row) show skin tissue with infiltration of epithelioid cells involving the subcutaneous tissue (x20 right, x10 left). The infiltrate is composed of cells with irregular to folded nuclei with prominent nuclear grooves, vesicular chromatin, distinct nucleoli, and moderate amounts of pale eosinophilic cytoplasm. Frequent admixed eosinophils are seen. Immunohistochemical studies (lower row) demonstrate that the lesional cells are strongly positive for CD1a (right) and S100 (left).

Itchy erythematous lesions were incidentally discovered over her scalp that occasionally appeared on her cheeks. These skin lesions were multiple, discrete, non-tender, non-scaly, erythematous, non-blanching papules over the scalp, forehead, and breast (Figure 1A).

The patient reported that these lesions started to appear two years earlier, but she did not seek medical advice. Otherwise, her past medical history was irrelevant. She did not experience headaches, vomiting, diplopia, weight loss, or bony aches. Polyuria was confirmed with the assessment of 24-hour urine output that exceeded 3 L/day. Further lab assessment showed a plasma sodium (Na) level at 139 mEq/L, plasma potassium level at 4.3 mEq/L, serum creatinine at 0.8 mg/dl, blood urea at 21 mg/dl, blood urea nitrogen (BUN) at 9 mg/dl, fasting blood sugar at 90 mg/dL, two-hour post-prandial level at 130 mg/dL, and glycosylated hemoglobin (HbA1c) at 6%. Twenty-four-hour urine osmolality was ˂ 300 mOsm/kg H2O (205 mOsm/kg H2O), which resolved with water diuresis. A water deprivation test with desmopressin administration was performed to assess the cause of polyuria associated with water diuresis, and normal plasma Na level was measured (differential diagnosis of primary polydipsia, central diabetes insipidus (CDI), or nephrogenic diabetes insipidus), which established a diagnosis of CDI [18].

She started desmopressin 0.1 mg twice daily, and the dose was adjusted according to the frequency of polyurea and nocturia. Further hormonal profile to assess for pituitary function showed serum prolactin level at 34.07 ng/ml, and within-normal values of 9 am cortisol level (12.36 µg/dl), follicle-stimulating hormone (FSH) level (5.63 mlU/ml), luteinizing hormone (LH) level (9.12 mlU/ml), free triiodothyronine (FT3), free thyroxine (FT4), and insulin-like growth factor 1 (IGF-1) (106 ng/ml).

**Figure 2:**
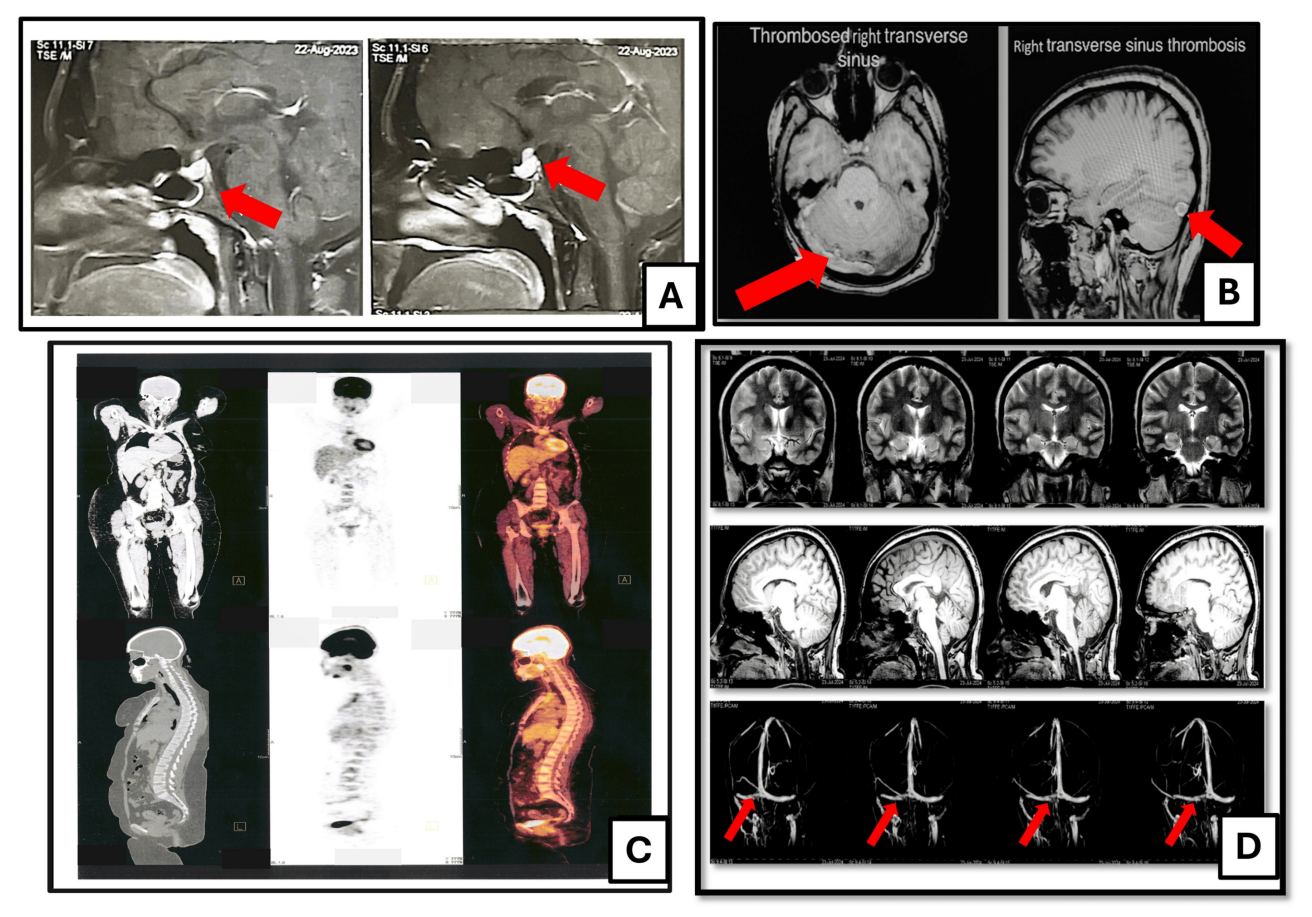
Radiological assessment of case 1. (A) MRI of the sella showing bulky posterior pituitary gland harboring a 7 mm hypo-enhancing lesion (red arrow), associated with thickened pituitary infundibulum reaching 6 mm in anteroposterior (AP), sagittal section (SS), and craniocaudal (CC) dimensions and 5 mm in SS dimension, representing suprasellar extension. (B) The right transverse and sigmoid sinuses show a loss of normal signal and no flow on post-contrast sequences (red arrows). (C) PET/CT scan showing prominent palatine tonsil, adenoid, and thymic tissue showing fluorodeoxyglucose uptake. (D) MRI of the brain (upper and middle rows) with magnetic resonance venography (MRV) (lower row) showing bilaterally distal transverse sinus stenosis (red arrows), focal on the right side and long segmental on the left side, followed by reconstitution at sigmoid venous sinus.

MRI of the sella turcica revealed posterior pituitary macroadenoma dimensions of 7 × 6 mm with a bulky hyper-enhancing pituitary stalk (Figure 2A). LCH was the provisional diagnosis, so the patient was referred to a dermatologist, and a punch biopsy from the forehead was done for histopathological evaluation. Skin biopsy revealed diffuse dermal infiltrate of histiocytes, lymphocytes, and eosinophils. The histiocytes had a slightly folded nucleus and eosinophilic cytoplasm. The histopathology was indicative of LCH. Further immunohistochemistry (IHC) immunophenotyping on skin biopsy was positive for CD1a, S100, CD68, and cyclin D-1 (Figure 1B) [7]. Together, the presence of CDI and the associated skin lesions confirmed the primary diagnosis of LCH (Table 1).

**Table 1:** Summary of the clinical aspects and differences of both cases. LCH: Langerhans cell histiocytosis; CD: cluster of differentiation; CDI: central diabetes insipidus; CNS: central nervous system; CNS-LCH: central nervous system-Langerhans cell histiocytosis; LN: lymph node; MS-LCH: multiple system-Langerhans cell histiocytosis; RO: risk organ; MRI: magnetic resonance imaging; RDD: Rosai-Dorfman disease.

	Case 1 (LCH)	Case 2 (RDD)
Age	32 years	38 years
Sex	Female	Male
Clinical presentation	CDI, itchy skin, lesion	Retro-orbital mass (proptosis), multiple skin nodules
Classification [5]	L group	R group
Clinical classification	MS-LCH, RO	Extra-nodal systemic RDD disease
	Skin	Skin
	LN CNS: hypothalamic-pituitary axis (CNS-LCH)	CNS
Immunohistochemistry		
CD1a	Positive	Negative
S100	Positive	Positive
CD65	Positive	Not tested
CD68	Not tested	Positive
Cyclin-D1	Positive	Not tested
CD3	Not tested	Positive
CD20	Not tested	Positive
Radiological assessment		
Method	MRI	MRI
Mass location	Posterior pituitary macroadenoma	Right retro-orbital
Description	7 × 6 mm with bulky hyper-enhancing pituitary stalk	Right para-sellar extra-axial enhancing lesion encasing the right internal carotid artery and extending through the foramen rotundum into the pterygopalatine fossa, inferior orbital fissure, and intra-orbital canal
Treatment		
	Vinblastine	Methotrexate and steroids
	Dexamethasone	
	Oral methotrexate	Rituximab
	Intrathecal methotrexate	Vinblastine

Further radiological work-up by whole-body PET/CT revealed bulky palatine tonsils and adenoids with a maximum standard uptake value (SUVmax) of ˜10 and prominent thymic tissue at 3.5 × 2 cm with SUVmax of ˜4.5. No pulmonary infiltrations or bony osteolytic lesions were noted (Figure 2C). Further lab evaluation is shown in Table 2.

**Table 2:** Summary of the laboratory differences in both cases. ANA: antinuclear antibody; anti-dsDNA: anti-double-stranded DNA; BUN: blood urea nitrogen; LDH: lactate dehydrogenase; LH: luteinizing hormone; LCH: Langerhans cell histiocytosis; RDD: Rosai-Dorfman disease; WBC: white blood cells; FSH: follicle-stimulating hormone; ESR: erythrocyte sedimentation rate.

Laboratory tests			
	Case 1 (LCH)	Case 2 (RDD)	Ref.
Hemoglobin (g/dl)	12.3	13.5	12-15 (F), 13-16 (M)
Platelets (count/L)	370 x 10^9^	300 x 10^9^	150 x 10^9^-450 x 10^9^
WBC (count/L)	5.8 x 10^9^	7 x 10^9^	
Serum creatinine (mg/dl)	0.8	0.7	0.5-1.1
BUN (mg/dl)	9	13	5-20
Plasma sodium (mEq/L)	139	141	135-145
Plasma potassium (mEq/L)	4.3	3.9	3.5-5
24h urine Osm (mOsm/kg)	<300	NA	500-850
Serum prolactin ng/ml	34.07	NA	<20 (M), < 25 (non-pregnant F)
9 am cortisol µg/dl	12.36	NA	5-25
LH (mlU/ml)	9.12	NA	1.68-15
FSH (mlU/ml)	5.63	NA	4.7-21.5
ESR 1^st^ hour (mm/hr)	5	8	<15
LDH (U/L)	98	102	140-280
Virology	Negative	Negative	NA
ANA, anti-dsDNA	Not tested	Negative	NA

The patient was started on dexamethasone (8 mg daily), oral methotrexate (10 mg weekly), and intrathecal methotrexate (15 mg weekly) and showed a very good response in terms of her skin lesions and sellar mass size. There was a remarkable improvement in her symptoms as well.

The patient was started on vinblastine 10 mg IV weekly bolus, with dexamethasone 16 mg IV weekly, in addition to oral methotrexate 15 mg once weekly, all for 12 weeks in addition to intrathecal (dual therapy) with methotrexate 12 mg + 2 mg dexamethasone.

Later on, 12 hours post lumbar puncture (LP), she developed a severe persistent headache that lasted for seven days. It was not improved by IV fluids, analgesia, and dexamethasone as a management of post-LP headache. After neuropsychiatry consultation, the CT of the brain requested was unremarkable, and an MRI of the brain with magnetic resonance venography (MRV) showed right transverse and sigmoid sinuses thrombosis (Figure 2B). She started anticoagulation with rivaroxaban 15 mg twice daily for 21 days, then 20 mg once daily for three months.

One month following the start of vinblastine, the patient showed marvelous improvement with the complete disappearance of her skin lesions (scalp, forehead, and breast). Three months later, the patient underwent a follow-up by PET/CT scan that showed significant improvement with complete resolution of the previously noted thymic tissue and metabolic regression of the previously noted palatine tonsils and adenoid with SUVmax of 8 (previously was 10). Also, a follow-up MRI brain with MRV showed recanalization of the previously noted right transverse and sigmoid sinus thrombosis (Figure 2D). 

The patient is planned to continue treatment with vinblastine 10 mg once weekly for a further three months as a single agent, as the patient is not tolerating methotrexate, and steroids plus anticoagulation with rivaroxaban 20 mg once daily for a further three months to prevent recurrent dural venous thrombosis.

Case 2

A 38-year-old male patient presented with recurring skin nodules of the back and lower extremities that first appeared in 2020. He sought medical advice and underwent repeated surgical excisions; however, no additional evaluations were done. The past medication history of the patient was irrelevant.

**Figure 3:**
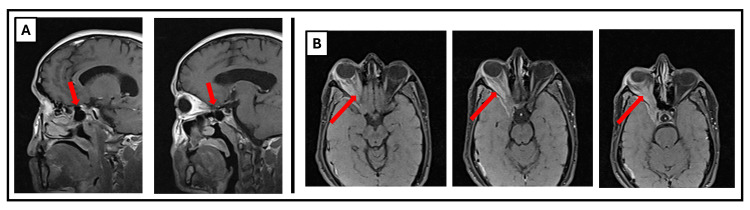
Radiological evaluation of case 2. (A) MRI of the brain sagittal sections with gadolinium showing right para-sellar extra-axial enhancing lesion encasing the right internal carotid artery and extending through the foramen rotundum into the pterygopalatine fossa, inferior orbital fissure, and intraorbital canal. (B) MRI of the brain transverse sections with gadolinium showing the para-sellar lesion extending via the right optic canal and superior orbital fissure to the right orbital apex, including the optic nerve and extraocular muscles, resulting in secondary proptosis.

**Figure 4:**
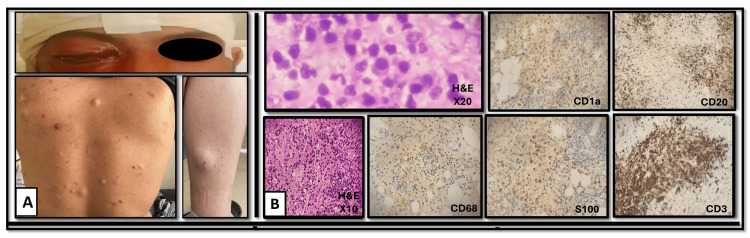
Gross and microscopic pathology of case 2. (A) The clinical presentation of the patient with Rosai-Dorfman disease with right eye proptosis and back covered with several skin lesions that were raised, soft in substance, non-tender, skin-colored, and varied in size, with the biggest being around 3 × 4 cm in diameter. Lastly, left calf nodule. (B) Hematoxylin & eosin (H&E) staining of the retro-orbital lesion showed chronic nonspecific inflammatory cells, including lymphocytes and plasma cells, Russell bodies, and histiocytes. Few elongated cells with oval nuclei were noted, interspersed by hemosiderin-laden macrophages. There were no meningothelial elements present. An image that suggests Rosai-Dorfman's illness. Immunohistochemistry for CD68 and S100 was performed, and both tests were positive. CD1a was negative, excluding Langerhans histiocytosis, and CD3 and CD20 were both positive, confirming the inflammatory nature of the tumor and excluding lymphoma.

In September 2021, he began to experience frequent headaches, right eye retro-orbital discomfort, and proptosis (Figure 4A). After evaluation by his ophthalmologist, an MRI of the brain was requested, which revealed right proptosis caused by an orbito-apical and spheno-orbital diffuse lesion, and a dilated right lateral ventricle (Figure 3A).

The ophthalmological assessment suggested an underlying autoimmune etiology and requested an electrophysiological study of the right optic nerve, which revealed a substantial optic nerve conduction deficit. Steroids were prescribed, but the patient was non-compliant with the treatment so its efficacy could not be assessed.

Six months later, there was a progression in his symptoms. MRI imaging of his brain, orbit, and paranasal sinus showed a grossly unchanged appearance from the previous one, with a right para-sellar extra-axial enhancing lesion encasing the right internal carotid artery and extending through the foramen rotundum into the pterygopalatine fossa, inferior orbital fissure, and intra-orbital canal. The lesion was seen extending via the right optic canal and superior orbital fissure to the right orbital apex, affecting the optic nerve and extraocular muscles, and resulting in secondary proptosis (Figure 3).

At this stage, malignancy was highly suspected, thus a thorough examination was requested. PET-CT revealed no substantial metabolic activity for the retro-orbital lesion. In October 2022, surgical removal of the tumor was done. Histopathological assessment of the excised mass revealed characteristics consistent with meningothelial meningioma (WHO grade I). Routine lab investigations also showed normal blood counts, renal function, and liver function tests.

The patient received no treatment until January 2023 when he presented with recurrent right eye pain and proptosis. MRI of the brain showed a right retro-orbital lesion, resembling hypertrophic pachymeningitis causing proptosis and encompassing the optic nerve (Figure 3B).

In June 2023, the patient underwent surgical resection of the retro-orbital lesion, and the pathology evaluation revealed a cellular lesion with an admixture of chronic nonspecific inflammatory cells, including lymphocytes and plasma cells, Russell bodies, and histiocytes. Few elongated cells with oval nuclei were seen interspersed by hemosiderin-laden macrophages. There were no meningothelial elements present. The whole clinical and pathological image suggested Rosai-Dorfman's illness, which was further emphasized by the positive immunohistochemical staining for both CD68 and S100 (Figure 4B) to differentiate it from the similarly appearing ECD [14]. The presence of slowly progressive disease and biopsy-proven chronic nonspecific inflammatory cells admixed with histiocytes positively stained for CD68 and S100 but not to CD1a established the diagnosis of extranodal and systemic (cutaneous and CNS) RDD.

After confirming the diagnosis in September 2023, the patient was referred for hematology consultation. On examination, he was vitally stable and seemed to be in good shape. His back was covered with several skin lesions that were raised, soft, non-tender, skin-colored, and varied in size, with the biggest being around 3 by 4 cm in diameter (Figure 4A). There was a single lesion on his left leg and another left preauricular one (Figure 4A). There was no lymphadenopathy or hepatosplenomegaly.

His lab tests revealed normal blood counts, including a hemoglobin of 13.5 g/dl, platelets of 300 x 109/L, and a white blood cell (WBC) count of 7 x 109 /L, with a normal differential count (Table 2).

The patient was started on a first-line treatment consisting of methotrexate 20 mg/m2 intramuscular (IM) weekly and dexamethasone 10 mg/m2 weekly for two weeks, then tapered to 5 mg/m2 weekly for two weeks, and 2.5 mg/m2 weekly for six months [14]. During treatment, the patient's skin lesions improved significantly. However, they gradually increased in size shortly after finishing treatment, therefore no imaging was ordered at this stage.

After progression on therapy, immunological re-evaluation was performed to rule out conditions associated with CNS masses. Antinuclear antibody (ANA) and anti-double stranded DNA (anti-dsDNA) were negative ruling out immune-related RDD. Virology tests for hepatitis C virus (HCV), human immunodeficiency virus (HIV), and hepatitis B surface antigen (HBsAg) were negative. Although RDD can be an immunoglobulin G4 (IgG4)-related disease, the presence of abnormal IgG4/IgG ratio is commonly associated with lung, liver, or gut involvement so immunoglobulin evaluation was not done. IHC for CD20 and CD3 was performed on the retro-orbital mass biopsy, and both were negative, ruling out lymphoma and suggesting a reactive character. CD1a was tested on the biopsy and was negative, ruling out Langerhans histiocytosis and confirming the diagnosis of RDD (Figure 4B).

In February 2024, the fast progression in less than four months prompted the start of a second line of therapy, rituximab 375 mg/m2 weekly for four weeks [14]. The treatment resulted in a clinical response with more than 50% regression in the skin lesions. Considering the high refractoriness and recurrence rates reported with rituximab and the aggressive nature of the disease [14], the decision at this stage was to add a third line of therapy. Accordingly, the patient was planned to receive vinblastine at a dose of 6.5 mg/m2 monthly for six months [19]. The patient had a very satisfactory clinical response after the first two doses, although some lesions were persistent on the back and lower extremities. The patient is planned to continue the remaining doses of vinblastine and to do a reassessment after six months by PET-CT.

## Discussion

Histiocytes, an important component of the innate immune system, grow and aberrantly activate in a broad range of confusing and unusual diseases known as HDs. Diagnosing and treating these disorders, which vary from the rare adult-onset ECD to the potentially deadly hemophagocytic lymphohistiocytosis (HLH) in children, is not straightforward [2].

HDs, despite their rarity, may present with a broad variety of symptoms, which lead to treatment challenges since they impact several organ systems. The management landscape for histiocytic illnesses is continually evolving as we get a better understanding of the underlying pathophysiology and introduce novel therapy options [20]. Here, we look at the complexities of these disorders, including difficulties in diagnosis and treatment, as well as the current status of practice in developing countries.

Our first case was a 32-year-old female, which falls within 25% of all LCH reports over the age of 15. There are no reported sex differences in LCH. Organ involvement in adults is quite similar to that in children; however, multisystem sickness may advance slowly and generate few symptoms initially. Recurrent tooth loss or dental cysts are common indicators of LCH. In addition, pituitary involvement might develop years before the beginning of other symptoms, as seen in our case. Alternatively, LCH might strike the upper lobes of the lungs, which are the most prevalent locations for micronodules and cysts in pulmonary LCH. Pneumothorax, dyspnea, and a dry cough are all possible signs of pulmonary LCH. Our patient has no lung or bone tumors that may mimic lytic metastases or myeloma [10,21].

A common location for CNS LCH is the hypothalamic-pituitary area, which causes anterior and posterior pituitary involvement, resulting in diabetes insipidus, growth hormone insufficiency, and thyroid function problems. In up to 63% of patients, MRI reveals tissue enlargement or cystic abnormalities in the pituitary stalk or pineal gland. The first case in point presented with diabetes insipidus, the most typical first symptom of CNS LCH [22].

Neither traditional therapy nor prospective studies have been conducted on adults with LCH. Because LCH may manifest in a wide variety of ways, the best course of therapy depends on the systems affected and the severity of the condition. Patients diagnosed with multiple systems (MS-LCH) or single-organ systems (SS-LCH) who have multifocal bone lesions are highly advised to undergo systemic treatment. There are currently no established therapies for this condition; however, similar to pediatric LCH, a regimen consisting of vinblastine and prednisolone may be the gold standard [23].

The intensity and duration of LCH therapy are condition-dependent. The prognosis is favorable, and treatment is usually not required for SS-LCH. Vinblastine, methotrexate, and prednisone are used to treat MS and SS LCHs with multifocal or significant anatomical regions [24]. After reviewing the literature and due to the unavailability of targeted therapy in Egypt, the BRAF-V600E mutation was not requested. Based on the clinical findings of this patient, she was considered as LCH-MS with CNS affection (CNS-LCH) without the risk of organ involvement [5]. Based on pediatric-inspired protocol [25], our patient started vinblastine 10 mg IV weekly bolus, with dexamethasone 16 mg IV weekly, in addition to oral methotrexate 15 mg once weekly, all for 12 weeks plus intrathecal (IT) (dual therapy) with methotrexate 12 mg + 2 mg dexamethasone [25].

First IT was given at the start of the treatment course. Later, 12 hours post-LP, she developed severe persistent headaches that lasted for seven days. It was not improved by IV fluids, analgesia, and dexamethasone as a management of post-LP headache. After a neuropsychiatry consultation, a CT of the brain was requested and was unremarkable. MRI of the brain with MRV showed right transverse and sigmoid sinus thrombosis (SST) (Figure 2B). She started anticoagulation with rivaroxaban 15 mg twice daily for 21 days, then 20 mg once daily for three months to prevent thrombosis recurrence following the German consensus-based (S2k) guidelines [26]. We found that it is possible that SST was caused by IT chemotherapy [27,28]. Another potential explanation for this presentation might be the relationship to LHC [29], or due to increased intracranial hypertension [30]. One month following the start of vinblastine, the patient showed clinical marvelous improvement with the complete disappearance of her skin lesions (scalp, forehead, and breast). She is planned for reassessment with whole-body PET/CT and a follow-up MRI of the brain with MRV three months later.

The second case illustrates another subtype of the histiocytic spectrum, which is RDD, in a 38-year-old male patient. Abla and his group reported that RDD often presents in younger age groups (mean age = 20.6); however, instances have been reported up to the age of 74. Males and persons of African descent have a greater frequency of RDD, whereas female Asians are more likely to have the cutaneous variety [14]. The predominant first symptom seen was the presence of subcutaneous lumps, which might be either painful or painless (Tables 1, 2) [15].

The rate of RDD misdiagnosis seems to be high as stated by Amoako et al. [31], especially with fine needle aspiration biopsies similar to what happened initially to the patient despite total excision of the mass. This raises the importance of IHC in making the diagnosis and characterization of different histiocytic disorders [32], which was missed in this patient’s first biopsy. An efficient IHC panel for recognizing and characterizing histiocytes includes CD68 or CD163, S100, CD1a, langerin, factor XIIIa, and cyclin D1 [33]. Applying most of this panel members verified both patients' diagnoses.

RDD treatment is tailored to the individual patient. Because 20% to 50% of nodal/cutaneous RDD patients have spontaneous remissions, monitoring is often recommended. This method is beneficial for those who have simple lymphadenopathy, asymptomatic cutaneous RDD, and few organs involved. Deciding the treatment plan for this patient started with surgical resection and histopathological evaluation. Also, resection may cure unifocal disease, and debulking may be required for upper airway obstruction, spinal cord compression, or large lesions affecting end organs [14]. Resection was followed by combined methotrexate, steroids, and vinblastine therapy. Chemotherapy is often reserved for refractory or relapsed patients; however, it is sometimes used as an initial treatment for disseminated or life-threatening disease [14]. Due to the suspected autoimmune nature of his initial eye affection, we used rituximab and it showed a fair clinical response [34,35].

High-intensity therapy substantially improved the survival of relapsed/refractory high-risk LHC and RDD patients. However, despite these improvements in survival and reactivation rate, the risk of persistent complications was only marginally diminished. Targeted medicines, particularly BRAF and MEK inhibitors, were proven beneficial in treating histiocytic illnesses in the recent decade, as the involvement of the MAPK pathway as a primary driver became increasingly apparent [36].

It is of utmost importance to raise medical community knowledge of these disease entities, particularly among different specialties. Indeed, promoting awareness of histiocytic disorders advances early identification and successful treatment. To achieve this objective, we started a multidisciplinary clinic that gathers different specialties from many healthcare facilities aiming at improving patient management and establishing a national registry for histiocytic disorders. By adopting the most updated treatment strategies, we will tailor the most appropriate methods for each patient's situation [14,15].

## Conclusions

Histiocytic disorders, a group of rare ailments characterized by abnormal histiocyte growth, provide a unique mixture of challenges and hopes for improving patient management. HLH, ECD, and LCH are only a few of the complex immune system illnesses that underscore the critical need for novel research and targeted treatment methods. Despite their rarity, they do have a significant impact on both patients and their families. These disorders often involve several systems, necessitating multidisciplinary and comprehensive approaches. More precise diagnostic modalities and therapies are on the way, due to the advances in genetic and molecular research, which shapes the way for better management and outcomes. Research prospects in this arena are arising, and progress in the field can be accomplished when researchers, clinicians, and patient organizations collaborate to revolutionize diagnostic techniques and develop novel therapeutics.
